# "Idiopathic" mental retardation and new chromosomal abnormalities

**DOI:** 10.1186/1824-7288-36-17

**Published:** 2010-02-14

**Authors:** Cinzia Galasso, Adriana Lo-Castro, Nadia El-Malhany, Paolo Curatolo

**Affiliations:** 1 Department of Neuroscience, Paediatric Neurology Unit, "Tor Vergata" University of Rome, Italy

## Abstract

Mental retardation is a heterogeneous condition, affecting 1-3% of general population. In the last few years, several emerging clinical entities have been described, due to the advent of newest genetic techniques, such as array Comparative Genomic Hybridization. The detection of cryptic microdeletion/microduplication abnormalities has allowed genotype-phenotype correlations, delineating recognizable syndromic conditions that are herein reviewed. With the aim to provide to Paediatricians a combined clinical and genetic approach to the child with cognitive impairment, a practical diagnostic algorithm is also illustrated. The use of microarray platforms has further reduced the percentage of "idiopathic" forms of mental retardation, previously accounted for about half of total cases. We discussed the putative pathways at the basis of remaining "pure idiopathic" forms of mental retardation, highlighting possible environmental and epigenetic mechanisms as causes of altered cognition.

## Introduction

Mental retardation (MR) is a variable, heterogeneous manifestation of central nervous system dysfunctions, occurring in 1-3% of general population [1]. MR represents one of the most frequently diagnosed disabling condition in our society, and a lifelong disability characterized by impairment of cognitive and adaptive skills.

The aetiology is very heterogeneous and, unfortunately, in about than one-half of cases the cause of MR is still elusive [2]. Anything that damages and interferes with the growth and maturation of the brain can lead to MR, and this might happen before, during or after the birth of the child (complications of pregnancy/birth, toxics, malnutrition, trauma, infections, understimulation). Moreover, genetically determined MR aetiology (comprising chromosomal aberrations, single-gene disorders, and other genetic conditions) account by itself for 17 to 41% of cases, depending of the different techniques of analysis [2].

Several syndromes (such as Down, Rett syndrome, and other well known conditions) should be easily suspected because of their association to specific dysmorphisms, behavioural peculiarities, and multiple congenital abnormalities. However, a consistent percentage of children with genetic MR do not present a recognizable phenotype striking of a well-recognizable syndrome.

With the advent of novel genetic techniques, several new cryptic chromosomal aberrations have been discovered in last few years [3,4], and a consistent number of MR cases, previously considered "idiopathic" forms, are now classified as syndromic conditions with clinical recognizable phenotypes [5]. Microarrays techniques (such as array-Comparative Genomic Hybridization, array-CGH) revealed submicroscopic aberrations in 5-17% of MR patients with normal results from prior conventional cytogenetic testing [6], and higher-density platforms (such as Single-Nucleotide Polymorphism array, SNP array) provided to increase diagnosis in about 6% of cases evaluated by lower-density oligonucleotide arrays [2].

Determining a specific etiologic diagnosis is central to understand the nature of the problem, providing answers to questions regarding prognosis, recurrence risks, directing specific therapies, and achieving meaningful inclusion of individuals with disability into society.

### Search strategy and selection criteria

Information in this review is mainly based on peer-reviewed medical publications of syndromic conditions from 2005 to 2010 (PubMed). Selection criteria are the novelty and importance of studies, and their relevance to Paediatricians. Search terms included "idiopathic mental retardation", "mental retardation", "cryptic chromosomal abnormalities", and "array-CGH". Only articles published in English were reviewed. All articles were read by the authors and references were reviewed to identify any additional relevant studies.

## Clinical Approach

The clinical approach of a child with MR is a key moment to provide a definitive diagnosis, and requires some exhaustive and comprehensive evaluations of the patient.

First of all, a three-generation pedigree should be done, and a detailed pre-, peri- and postnatal history is mandatory. A dysmorphic child may be at risk from the stress of birth, and later delay may be erroneously attributed to birth injury [7]. A careful developmental history, with emphasis on milestones, formal assessments and behavior, is also required. Medical records should be sought or requested to validate any diagnosis of malformations. An accurate EEG study and/or brain MRI are sometimes sufficient to suspect several well-known and relatively common disorders (such as Rett syndrome, Angelman syndrome, neurocutaneous syndromes, etc.) [8,9]. The degree of MR is an important indicator: the so called "chromosomal" phenotype, which is well known to accompany larger aberrations, is frequently characterized by moderate-severe MR associated to one or more of major signs, including congenital malformations. The behavioral phenotype is also distinctive for several well-known syndromic conditions, such as Williams syndrome, Angelman syndrome, Prader-Willi syndrome, and so on [10].

Finally, the physical examination of the child is crucial for a "gestaltic" diagnosis: sometimes the syndromic condition can be instantaneously suspected by recognition of "handles", based on past clinical experience. Hovewer, phenotypic expression among patients with well-recognized microdeletion or microduplication syndromes may vary on the basis of different sizes of genomic alterations, and of individual differences in the rest of the genome. Unfortunately, in many cases the MR is the unique and unspecific sign present in the patient, with lack of major hallmarks. When present, minor anomalies of the face (such as hypo-hypertelorism, unusual ear conformation, multiple hair whorls, etc.), hands, genitalia, and skin should be noted and supplemented by objective measurements. Abnormalities in head size, growth parameters, and neurologic signs should be carefully investigated.

The phenotype can also vary during the time, it should be useful to collect photos and/or videos of patients at different ages, also because the amount of controls in our experience often affects the probability to define the etiology.

## Genetic Approach

Genetic abnormalities are the most common identifiable cause of unexplained MR [11], but conventional karyotyping is unable to detect imbalances smaller than about 3-5 Mb [12]. Smaller chromosomal abnormalities can be identified with fluorescent *in situ *hybridization (FISH) or multiplex ligation-dependent probe amplification (MLPA) techniques, confirming a clinical suspicion of well-known microdeletion/microduplication syndromes (i.e. Williams syndrome, Velocardiofacial/DiGeorge syndrome, etc.) or analysing subtelomeric regions of all chromosomes. The combined analysis of karyotype and subtelomeric regions, using FISH or other molecular techniques, have allowed the detection of chromosome abnormalities in about 5-10% of these patients [11,12]. The newer chromosome microarray or comparative genomic hybridization technique (array-CGH) is an efficient manner to approach a case of MR. It does not require an expert clinician to suspect a specific diagnosis, and may cover the entire genome or targets known pathologic loci in an unique test, identifying deletions and/or duplications with a higher degree of sensitivity. This new technique has revealed submicroscopic chromosome aberrations in MR patients with normal results from prior cytogenetic analyses with detection rates as 5-20% [6,13]. However, array-CGH is incapable of detecting balanced rearrangements of chromosomal material (including reciprocal translocations and inversions), which are expected to occur in about 0.75% of all MR patients [14]. Moreover, the interpretation of microarray data in MR is complicated by the discovery of areas of DNA segment longer than 1 kb, with a variable copy number compared with a normal reference genome, called Copy Number Variations (CNVs) [15]. CNVs are associated to a pathological phenotype when one or more dosage-sensitive genes inside the rearranged region are altered. However, CNVs are not considered pathologic in all cases, because they appear to be conserved across primate species and may be responsible of individual diversity and human evolution. In a single individual, it is possible to detect even > 1000 non-pathological common CNVs [16], needing the comparison with unaffected control cohorts and parental tests [17]. The detection of a relatively large, rare, *de novo *CNV in an affected patient is strongly indicative of pathological significance, and is present in about 10% of cases of MR with normal chromosome analysis [1,18]. CNVs should be considered causative of the condition when: 1) CNVs overlap with regions known to cause well delineated MR syndromes; 2) CNVs include the critical region of a syndrome or causative genes; 3) the phenotype of the patient is consistent with the syndrome's features [2].

## Combined Diagnostic Algorithm

**Figure 1:**
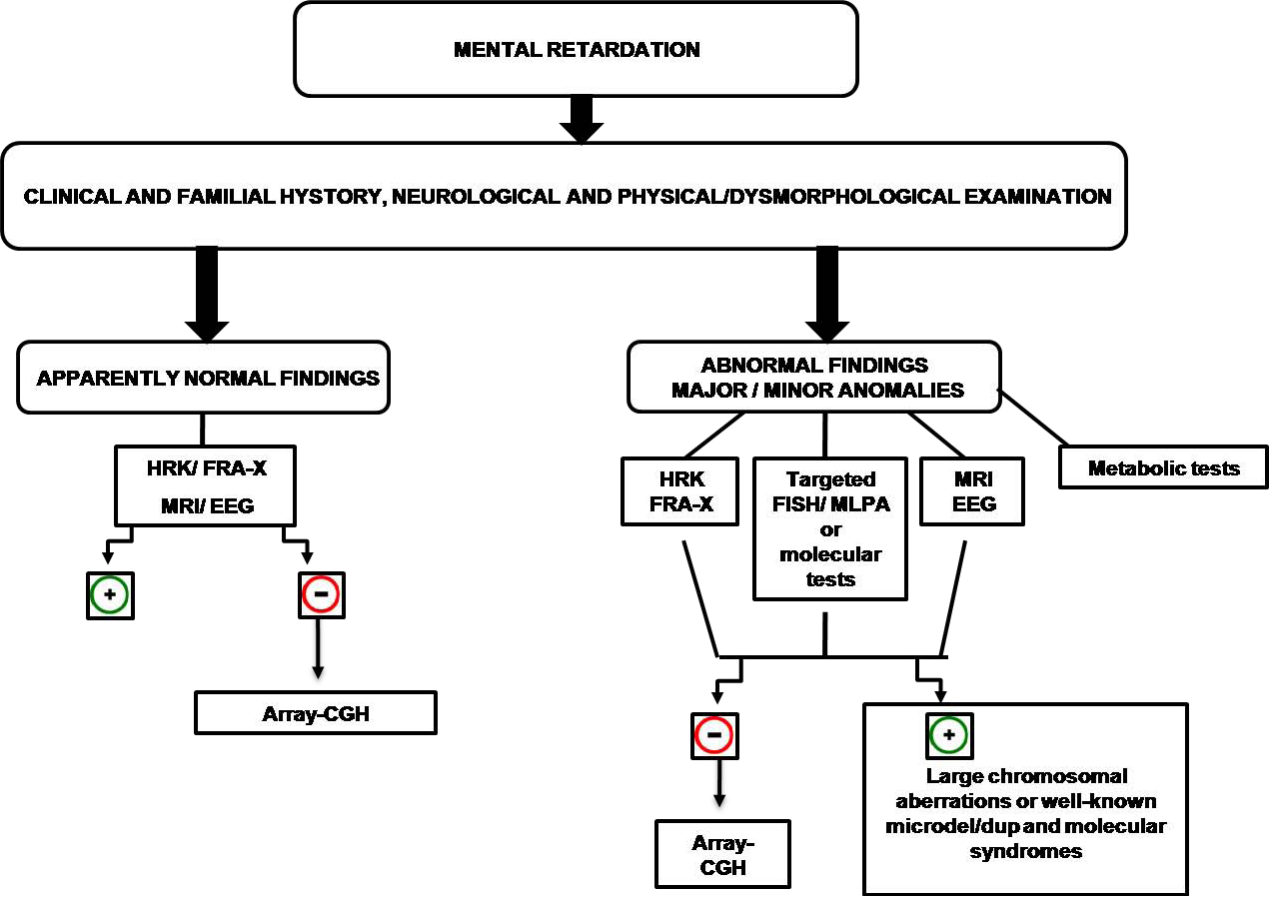
**Diagnostic algorithm proposed for unexplained MR cases of suspected genetic origin**. Array-CGH = array-Comparative Genome Hybridization; FISH: Fluorescent *In Situ *Hybridization; Fra-X: Fragile × syndrome molecular analysis; HRK: high-resolution karyotype; microdel/dup: microdeletion/microduplication; MLPA: Multiplex Ligation-dependent Probe Amplification.

A possible diagnostic algorithm which can be useful in the evaluation of a child with unexplained MR and suspected genetic condition [8,9] is illustrated in the Figure 1. After a detailed anamnesis assessed by three-generations pedigree analysis, a comprehensive cognitive, behavioral and physical examination (clinical and instrumental) is mandatory. Depending on the suspected or not-suspected diagnosis, the work-up of a patient can follow two different pathways, guiding to decisions regarding laboratory testing and imaging studies. On the basis of the presence of dysmorphic signs and/or major abnormalities (such as multiple congenital defects), high-resolution karyotype, fragile-X DNA analysis, targeted FISH/MLPA, or metabolic tests should be considered. EEG and/or MRI are also useful. Finally, array-CGH is indicated when above-mentioned tests are negative.

When the child has an apparently normal phenotype, after high-resolution karyotype, fragile-X molecular tests, EEG, and MRI studies, array-CGH analysis should be directly performed to exclude a cryptic chromosomal aberration.

## Chromosomal Abnormalities

Recent developments in array technology have strongly changed the genetic approach to MR, combining the whole-genome analysis of karyotyping technology and the targeted high-resolution of FISH test. Genomic microarrays have a resolution 10-10000 times higher than that of conventional karyotyping, identifying rare, *de novo*, submicroscopic interstitial imbalances or CNVs in about 5-20% of cases of idiopathic MR and multiple congenital abnormalities, depending on the clinical selection of patients [1,12]. The increased identification of novel microdeletion/microduplication syndromes is based on an accurate genotype-phenotype correlation, characterized by the association of similar chromosomal aberrations and overlapped clinical presentations between affected patients.

The ability to recognize pathological gestalts and/or behaviors has already led to a significant improvement in the diagnostic yield in patients with MR. Several example of relatively common, novel well-delineated syndromes are discussed in this review. Salient phenotypical traits of each syndrome are also synthesized in Additional file 1.

### 1p36 microdeletion syndrome

**Figure 2:**
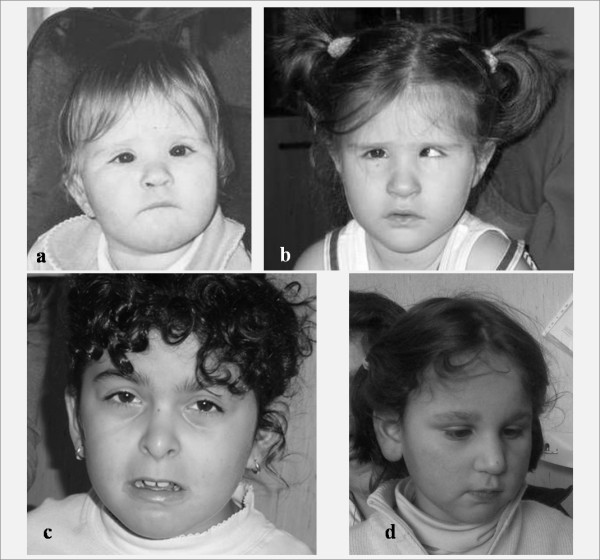
**Pictures of three patients with mental retardation and dysmorphisms with a genetic diagnosis**. **a) ***de novo *1p36 deletion in a 22 months old girl; **b) **The same patient at 3 years of age. Note prominent forehead, very straight eyebrows, epicanthus, deep-set eyes, flat nasal bridge, and thin lips; **c) ***de novo *2q37.1 deletion in a 7 years old girl. Note bushy eyebrows, horizontal palpebral fissures, flat nasal philtrum with prominent columella, thin upper lip, high palate, microretrognatia, and rather asymmetrical ears; **d) **22q11.2 duplication inherited from affected mother, in a 4 years old girl. Note high forehead, sparse eyebrows, short and downslanting palpebral fissures, hypertelorism, bulbous nose, pronounced philtrum, fullness of jowls, and large and simplified ears with protruding lobes.

Monosomy 1p36 is a well-described contiguous genes syndrome, considered as the most common terminal deletion observed in humans, accounting for 0.5-1.2% of idiopathic MR [19,20]. Dysmorphisms are very remarkable and distinctive of this condition: microcephaly, large and late closing anterior fontanel, tower skull, prominent forehead, straight eyebrows, deep-set eyes, flat nasal bridge with midface hypoplasia, abnormal ears, brachydactyly/camptodactyly, and short feet (Figure 2a, b). Severe hypotonia, seizures, oropharyngeal dysphagia, and heart defects are also common [20]. MR of any degree, mostly moderate to severe, is present in all individuals, associated to a severe speech impairment and poor coordinated movements control [19]. Subtelomeric FISH analysis, targeted FISH analysis of chromosome 1 and/or array-CGH are needed to detect the 1p36 microdeletion. This syndrome should be considered in a young child with above-mentioned dysmorphisms, psychomotor delay and hypotonia, in particular when the language is poor or absent, and self-injuring behaviors, stereotypies, and hyperactivity are present.

### 2q23.1 microdeletion syndrome

This new syndrome has been identified by a-CGH in patients with severe MR and severe speech impairment, associated with microcephaly, coarse face, short stature, and epilepsy [21]. Frequently, the phenotype of syndrome includes stereotypic behaviors, altered sleep pattern and a broad-based gait, leading to the clinical impression of Angelman, Rett or Smith-Magenis syndromes [22]. Haploinsufficiency of *MBD5 *or *EPC2 *genes, included in the deleted genomic region, seems to be responsible of the typical phenotype [22,23].

### 2q37 deletion syndrome

Del(2q37) syndrome is now a well recognized disease, characterized by facial dysmorphic features (Figure 2c), developmental delay, hypotonia, epilepsy in 25% of cases, and major anomalies in about 30% [24,25].

Psychiatric conditions are frequently associated with del(2q37). Autism spectrum disorders is present in 24-35% of del(2q37) cases, but also severe speech delay, stereotypic movements, aggressive behavior, attention-deficit/hyperactivity disorder (ADHD), and obsessive-compulsive disorder are commonly observed [24]. In a child with MR and these neuropsychiatric disorders, the presence of facial dysmorphic traits and congenital defects, variably associated with short stature, obesity, brachydactyly, eczema, and hypotonia, should be considered highly suggestive of del(2q37). High resolution karyotype, FISH/MLPA or aCGH analyses are useful for diagnosis [25].

### 7q11.3 microduplication syndrome

Williams-Beuren syndrome (WBS), caused by deletion of a 1.4-1.5 Mb region located at 7q11.23, is among the most well-characterized microdeletion syndrome, but the reciprocal microduplication of this genomic region is less well described. The clinical phenotype of 7q11.23 microduplication seems to vary among patients, ranging from mild to severe MR [26]. The neurobehavioral phenotype is the opposite of WBS: instead of fluent expressive language, dup 7q11.23 patients show severe speech delay and only mildly impaired visuospatial skills [27]. Mild facial dysmorphisms (short philtrum, thin lips, and straight eyebrows), an increased incidence of heart defects, diaphragmatic hernia, cryptorchidism, and non-specific MRI brain abnormalities should orientate the clinician to make diagnosis [28].

### 15q13.3 microdeletion syndrome

The overall incidence of this aberration is about 0.3% of patients with "idiopathic" MR, considering it comparable to William and Angelman syndromes [29]. MR ranges from mild to moderate, and 15q13.3 deletion has also been recently associated to a higher predisposition to autism spectrum disorders, schizophrenia, other psychiatric disorders, and idiopathic generalized epilepsy or EEG abnormalities [30]. The syndrome has a highly variable intra- and inter-familial phenotype, with mild facial dysmorphisms, including hypertelorism, upslanting palpebral fissures, prominent philtrum with full everted lips, and short and/or curved fifth finger and short fourth metacarpals [31].

### 16p11.2 microdeletion syndrome

Microdeletion at 16p11.2 has recently been associated with autism in two different studies [32,33], but this syndrome is characterized by a variable phenotype, ranging from normal intelligence and mild dysmorphisms to severe cognitive impairment and minor/major congenital abnormalities. Facial features are characterized by flat and hypotonic facies, deep-set eyes, low-set and posteriorly rotated ears, and thin upper lip. Frequent ear infections, orofacial clefting, heart defects, and minor hand/foot anomalies have been described [34,35]. Expressive language disorder, dyslexia, and ADHD are also frequent [36].

### 17q21.31 deletion syndrome

This novel syndrome seems to have a prevalence of 1 in 16 000 individuals, and to be underestimated. In all patients mild-severe global psychomotor delay is noted from an early age, associated to hypotonia with poor sucking and slow feeding [37]. Facial dysmorphisms include long face high/broad forehead, upslanting palpebral fissures, anteverted and large ears, and the typically "tubular" or "pear shaped" nose with bulbous tip. Abnormality of hair pigmentation and texture, and in general, of ectodermal structures, are also observed [38]. The facial gestalt changes with age: in infancy the facial hypotonia, with an open mouth appearance, is predominant. With increasing age, the face becomes elongated and the tubular or pear shape form of the nose is more pronounced. In addition, patients with del 17q21.31 may have long fingers, nasal speech, and friendly disposition [37]. Other common features are cryptorchidism, epilepsy, hypermetropia, pectus excavatum, congenital heart defects, kidney and urologic anomalies, dislocation of the hips, and spinal deformities [38].

### 22q11.2 microduplication

Microduplication of 22q11.2 (dup22q11.2) has recently emerged as a new chromosomal syndrome (Figure 2c). Dup22q11.2 represents the reciprocal duplication of region deleted in Di George/Velocardiofacial syndrome (DG/VCFS), due to misalignment of low-copy repeats in this band. Although this syndrome shares features with DG/VCFS (heart defects, velopharyngeal insufficiency with or without cleft palate, hypernasal speech, and urogenital abnormalities), a clear genotype-phenotype correlation is not yet established [39]. Individuals with dup22q11.2 show normal intelligence or cognitive impairment of any degrees. Neurological and psychiatric disorders, including speech delay, learning disabilities, motor impairment, aggressiveness, anxiety, depression, autisms, ADHD, oppositional-defiant disorder, obsessive traits, and social interaction problems are frequently associated [39,40]. The microduplication is only detectable by interphase FISH, MLPA or a-CGH analyses, and the genetic test is recommended in children with central hypotonia, severe speech delay, learning disabilities/cognitive impairment, or psychiatric disorders including autism [40].

## Conclusions

It is generally assumed that severe forms of MR are thought to be due to larger chromosomal abnormalities or defects in single genes, in the majority of cases detectable with specific genetic tests. Paediatricians should be alerted by the presence of MR of unexplained origin associated with altered auxological parameters, multiple congenital defects, neurological and psychiatric signs, and/or minor dysmorphisms. However, many children who have MR and dysmorphisms often do not have major malformations, simply having an appearance that is unusual compared with the general population, and out of keeping with that of unaffected close relatives. In particular, mild forms of MR often lack suggestive clinical "handles", resulting from the interaction of multiple genes and non-genetic factors [41]. Despite the introduction of high-resolution platforms has facilitated the identification of emerging microdeletion/microduplication syndromes, in mild forms of MR making an etiological diagnosis is still very difficult. The quote of "idiopathic" forms of MR account for about half of total cases, probably due to combination of multigenic and environmental causes. The developing brain is more susceptible to insults by toxic agents than adults, because during the prenatal life several complex stages of organization and maturation must develop in a tightly controlled time. Moreover, the blood-brain barrier is not completely formed until the sixth month of intrauterine life, leading the developing brain exposed to toxins [42]. In addition, a mutation in a single gene (responsible a for genetic syndrome) may lead to epigenetic dysregulation [43], influencing transcription and/or silencing other genes. Epigenetic mechanisms play a central role in higher-order brain functions, influencing the capacity to modify, reorganize and remodel synaptic plasticity and networks during learning and memory formation, and in response to injury. Several well-known syndromes are caused by disrupted epigenetic mechanisms, such as Rett syndrome, and fragile × syndrome [44]. In these and others genetic conditions, as well as in environmental MR -associated disorders (e.g. fetal alcohol spectrum disorders or lead exposure) reduced dendritic complexity, and significant differences in dendritic spine numbers and morphological spine types have been observed [43,44]. However, epigenetic mechanisms are dynamic and potentially reversible, providing new pharmacological approaches to treat neurodevelopmental disorders. DNA-demethylating drugs and HDAC inhibitors are two promising examples of targeted epigenetic drugs [43]. The implementation of the so called "next generation sequencing" technologies (that allow the analysis of whole-genomes, transcriptomes and interactomes) could lead to detect single base mutations and structural variations, further broadening the possibility of diagnosis in "idiopathic" cases of MR. Understanding the pathological pathways underlying unexplained forms of MR represent a future challenge to increase both prevention and possible therapies.

## Consent

Written consents for publication of patients' pictures were obtained from their parents.

## Competing interests

The authors declare that they have no competing interests.

## Authors' contributions

ALC (Medical Doctor) drew the first draft with the assistance and contribution of NEM (Medical Doctor), and reviewed relevant articles on the literature under the supervision of CG (Associated Professor of the Department of Pediatric Neuroscience Unit) on clinical and neurogenetical aspects. PC (Director of the Department of Pediatric Neuroscience Unit) proposed and designed the study, and revised the final draft.

All authors contributed to the intellectual contents and approved the final version.

## Supplementary Material

Additional file 1 **Schematic overview of major features of novel syndromic conditions discussed in this paper. **For a more detailed description of each syndrome, refer to the text. (+) present feature; (-) absent feature; (+/-) inconstantly present/rare feature. ADHD: Attention-deficit/hyperactivity disorder; DD: developmental delay; IQ: intelligent quotient; MR: mental retardation. Click here for file
