# Supplementary material for: "Idiopathic" mental retardation and new chromosomal abnormalities
Source: Ital J Pediatr. 2010 Feb 14;36:17. doi: 10.1186/1824-7288-36-17 (PMC2844383; doi:10.1186/1824-7288-36-17)
Supplement: Additional file 1 — Schematic overview of major features of novel syndromic conditions discussed in this paper. For a more detailed description of each syndrome, refer to the text. (+) present feature; (-) absent feature; (+/-) inconstantly present/rare feature. ADHD: Attention-deficit/hyperactivity disorder; DD: developmental delay; IQ: intelligent quotient; MR: mental retardation. [file 1824-7288-36-17-S1.DOC]

**Additional file 1:** Schematic overview of major features of novel syndromic conditions discussed in this paper. For a more detailed description of each syndrome, refer to the text.

| **Syndrome** | **1p36 microdel** | **2q23.1 microdel** | **2q37 del** | **7q11.3 microdup** | **15q13.3 microdel** | **16p11.2 microdel** | **17q21.31 del** | **22q11.2 microdup** |
| --- | --- | --- | --- | --- | --- | --- | --- | --- |
| **Dysmorphisms** | Tower skull, prominent forehead, large and late closing anterior fontanel, straight eyebrows, deep-set eyes, flat nasal bridge with midface hypoplasia, abnormal ears, brachydactyly/camptodactyly, and short feet | Coarse facies | Prominent forehead, unusual eyebrow pattern, prominent columella, malformed or protruding ears | Short philtrum, thin lips, straight eyebrows, | Hypertelorism, upslanting palpebral fissures, prominent philtrum with full everted lips, and short and/or curved fifth finger and short fourth metacarpals | Flat and hypotonic facies, deep-set eyes, low-set and posteriorly rotated ears, thin upper lip | Long face high/broad forehead, upslanting palpebral fissures, “tubular” or “pear shaped” nose with bulbous tip, anteverted and large ears | High forehead, superior placement of eyebrows, hypertelorism, epicanthal folds, broad nasal bridge, bulbous nose, smooth philtrum, dysplastic large hears |
| **DD/MR** | Moderate - severe | Severe | All degrees | All degrees | Mild - moderate | Normal IQ – Severe DD/MR | All degrees | Normal IQ – Severe DD/MR |
| **Behavioral phenotype** | Speech delay, stereotypies, ADHD | Severe speech delay, stereotypic behaviors, altered sleep pattern | Autism or autistic like behaviors | Hyperactivity, aggressive outbursts, hyperphagia, speech delay | Autism or autistic features, schizophrenia, other psychiatric disorders | Expressive language disorder, autism, dyslexia, ADHD | Friendly behavior | Psychiatric disorders, speech delay, learning disabilities |
| **Epilepsy** | + | + | + / - | - | + | + / - | + | + |
| **MRI abnormalities** | + | - | + / - | + (non specific) | - | + / - | + / - | +/ - |
| **Craniofacial abnormalities** | Microcephaly/ brachycephaly; large anterior fontanel.  Oropharyngeal dysphagia | Microcephaly | - | Dolichocephaly | Non specific | Macrocephaly | + | + |
| **Scheletrical abnormalities** | + / - | - | Albright phenotype, brachidaptily | + / - | + / - | + | + | - |
| **Gastrointestinal abnormalities** | + | - | + | + | - | + / - | + | + / - |
| **Genitourinary abnormalities** | - | - | + | + | - | - | + | + |
| **Cardiac defects** | + | - | + | + | - | + / - | + | + |
| **Others** | Hearing impairment, growth impairment, hypotonia, muscle hypotrophy, poor coordination | Broad-based gait | Hypotonia | - | Oromotor dyspraxia | Frequent ear infections | Early severe hypotonia, nasal speech, abnormal ectodermal structures | Joint hyperlaxity, hypotonia, motor impairment |

(+) present feature;

(-) absent feature;

(+/-) inconstantly present feature/ rarer feature.

ADHD: Attention-deficit/hyperactivity disorder; DD: developmental delay; IQ: intelligent quotient; MR: mental retardation.
